# Cystic Fibrosis-Associated Bilateral Pseudomucocele - Case Report

**DOI:** 10.1016/S1808-8694(15)31186-1

**Published:** 2015-10-19

**Authors:** Karla Palma Portes, Silvio Antonio Monteiro Marone, Elder Yoshimitsu Goto, Cleber Palma, Maria Renata Macca Ferreira Jorge, Denilson Antonio Cavazzani Szkudlarek

**Affiliations:** 1 2nd Year-Resident - Santa Marcelina Hospital; 2 PhD. Professor of Otolaryngology - FMUSP, Full Professor of Otolaryngology - PUC Campinas Medical School. Coordinator of the Medical Residency in Otolaryngology - Santa Marcelina Hospital; 3 PhD Student – Department of Otorhinolaryngology – Medical School – University of São Paulo (FMUSP), Professor of Otolaryngology - FMUSP, professor in charge of rhinology of the Residency Program in Otorhinolaryngology - Santa Marcelina Hospital; 4 3rd Year-Resident - Santa Marcelina Hospital; 5 3rd Year-Resident in Otolaryngology; 6 MD. ENT - Hospital Santa Marcelina Hospital- SP

**Keywords:** cystic fibrosis, pseudomucocele, ct scan of the paranasal sinuses

## Abstract

**Summary:**

Cystic fibrosis, also known as mucoviscidosis, is a monogenetic disorder that is presented as a multisystemic disease. The incidence is approximately 1: 2500 live births. The pathophysiologic mechanism is a qualitative change in all exocrine secretions of the body. An increased viscosity of those secretions leads to stasis and mechanical obstruction, resulting in an impaired function of secretory and target organs. Nose and sinuses are involved due to abnormal mucociliary clearance, responsible for chronic rhinosinusitis, nasal polyps and sinus pseudomucocele. **Objective**: show a rare case of bilateral pseudomucocele in a child with cystic fibrosis. **Case description**: M.F.B.R., 2 years old, male, with nasal obstruction and recurrent pulmonary infections. Clinical findings were copious nasal secretion and posterior nasal drip. The CT scan of the paranasal sinuses showed an image that was suggestive of pseudomucocele, with opacification of maxillary and ethmoid sinuses. The sweat test presented meaningful results. We preferred surgical treatment, after patient hospitalization, to control the pulmonary manifestations. The child presented improvement of nasal obstructive symptoms.

**Conclusions:**

Pseudomucocele is a disease that has been increasingly included in the routine of the differential diagnoses since CT scans became part of sinus disease semiology. Patients with pseudomucoceles have enjoyed relevant increases in their survival, thanks to current treatment modalities.

## INTRODUCTION

Cystic fibrosis is a monogenetic disorder that manifests itself as a multisystemic disease. The first signs and symptoms are usually seen in childhood; however, almost 3% of patients are diagnosed at a more mature age.^1^ It is a recessive autosomal disease accruing from mutations in a gene located on chromosome^7^. Prevalence varies according to the ethnical origin of the population. It is identified in 1:2500 live births in the caucasian population of North America and Europe; in 1:17000 live births in the African-American population and in 1:90000 live births in the Asian population.^2^ The most common mutation in the cystic fibrosis gene is a deletion of one pair of three bases that results in the lack of phenylalanine in position 508 of the amino acids of the protein produced by the gene, known as cystic fibrosis transmembrane regulator (CFTR).^3^

Both the nose and the paranasal sinuses are involved due to an abnormal mucociliary clearance; however, the most affected organs are the pancreas, liver and lungs. Mechanical obstruction of the common duct and the choledoch are responsible for malnutrition and later pancreatic fibrosis and liver cirrhosis. In the lower respiratory tract, stases of a viscous mucous causes bronchiectasias with a chronic contamination by multiresistant germs and later on by fungi. The progressive worsening in the respiratory function causes severe cardio-respiratory failure.^14^

Rhinosinusitis is not an uncommon disorder present in children and adults with cystic fibrosis. Wang et al. observed that mutations in the gene responsible for cystic fibrosis predispose patients to chronic rhinosinusitis.^5^ This sinuses disease does not impact the life expectancy of these individuals; however, it increases disease morbidity, with a worsening in the life quality of such patients and it may even deteriorate their pulmonary status.6 This viscous mucous stasis is responsible for intraluminal retention, ostium obstruction, chronic sinus inflammation and infection. Nasal polyposis was the first nasal manifestation described.^7^ Other manifestations, seen mainly through radiologic studies, include changes to the uncinate process, chronic sinusitis without polyposis, destruction of lateral nasal wall bony structures and pseudomucoceles. Coste et al. described the pseudomucocele for the first time in 1995. He observed in paranasal sinuses CT Scans of children with cystic fibrosis the presence of images with a hyperdense and heterogenous core, having the periphery in hypodensity. The maxillary sinus is affected in 100% of the cases, the ethmoid in 55% and the sphenoid in 60% of the cases. The intraoperative finding in these patients show a thick inflammatory capsule, following the sinus wall contour and a viscous secretion inside.^8^

## CASE REPORT

M.F.B. R., 2 years and one month old male, brown. Was referred to the Otorhinolaryngology ward of the Santa Marcelina Hospital to undergo chronic nasal obstruction assessment. The mother reported that the child presented with chronic oral breathing since birth, snored heavily at night and frequently had a runny nose with thick yellowish secretion. She also reported that he had repeated pulmonary infections, and numerous hospital stays due to respiratory disorders, besides delays in growth and weight gain. There was no family history of atopy or respiratory disorders. The child frequently used systemic steroids. Vaccination was adequate for the child's age.

At the general physical exam, the child was in regular general health status, cushingoid facies, dyspneic, noisy breathing, non-cyanotic, hydrated and feverless. Weight: 10 kg; Height: 80 cm. During otolaryngological exam we noticed opaque tympanic membranes with increased vascularization. Anterior rhinoscopy showed abundant yellowish secretion, and no nasal tumors. At oroscopy, we detected post-nasal dripping. Chest exam showed a narrow and triangular-shaped chest and wishbone movement at breathing, snores and murmurs during pulmonary auscultation. A naso-gastric tube was inserted without major difficulties through both nasal cavities, clinically ruling out stenosis or choanal atresia. Nasofibroscopic exam revealed abundant bilateral yellowish nasal secretion. We did not find polyps, nasal tumors or severe adenoid hypertrophy. While we waited for the image study results, the child received antibiotics, steroids, nasal flushing and inhaled bronchodilators. Paranasal sinus CT Scan showed a pseudomucocele-suggestive image, with veiling of the maxillary and ethmoid sinuses bilaterally by some soft tissue mass. We also ordered an MRI in order to better assess the paranasal sinuses content, confirming the idea of it being a pseudomucocele. In order to confirm the diagnosis of cystic fibrosis, we measured sodium and chlorine ions present in sweat, which varied significantly (Sodium:155mEq/l; Chlorine:185-mEq/l). The sweat test was repeated and confirmed. We deem the test positive when the chlorine ion concentration in the sweat is greater than 60mEq/l in children and greater than 80mEq/l in adults.

**Figure 1:**
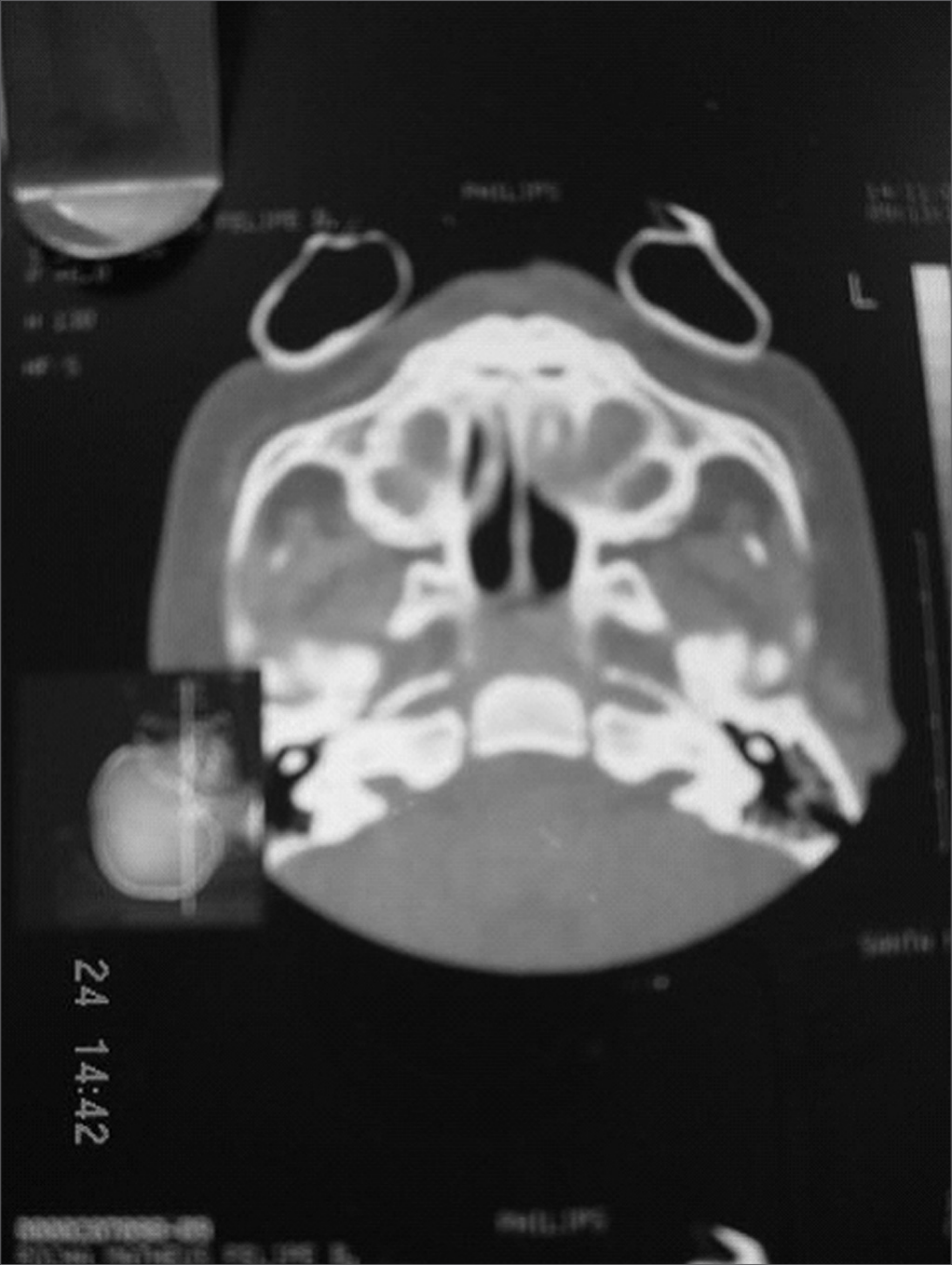

The pseudomucocele bulges against the lateral nasal wall, narrowing the nasal cavity, thus reducing its permeability, causing important respiratory discomfort for the patient. We then decided for the surgical treatment in order to mitigate the upper respiratory obstruction. However, the child's pulmonary condition did not respond to clinical treatment. He was admitted to the hospital in order to control his respiratory disorder and was treated by a multidisciplinary team made up of an otorhinolaryngologist, pediatricians and anesthesiologists. When he was better enough to undergo surgery, we performed endoscopic maxillary antrostomy and ethmoidectomy through the inferior meatus and middle meatus, respectively; without complications. During surgery we found an inflammatory mucosa, purulent secretion in large amount and the bulging of the nasal cavities lateral walls. Polyps were not found. The child had an improvement in his obstruction already in the first days following surgery. Histology of the paranasal sinuses' content revealed inflammatory tissue and hyperplasia of the mucous glands.

## DISCUSSION

This relationship between chronic rhinosinusitis and cystic fibrosis has been broadly studied and well documented in papers published in recent decades.^4^, ^5^, ^6^, ^7^, ^8^ The most frequent manifestation is nasal polyposis associated to chronic sinus disease, and the polyps may be seen even in children with cystic fibrosis. This condition is also more frequently seen in male patients.^4^,^9^

In the case hereby described, we did not see nasal polyps during the endoscopic exam. We were only able to see a viscous and yellowish secretion. CT scan showed an image very much suggestive of pseudomucocele, also deemed a frequent manifestation in series studies of nasal disorders in cystic fibrosis patients.^4^,^8^

**Figures 1 and 2:**
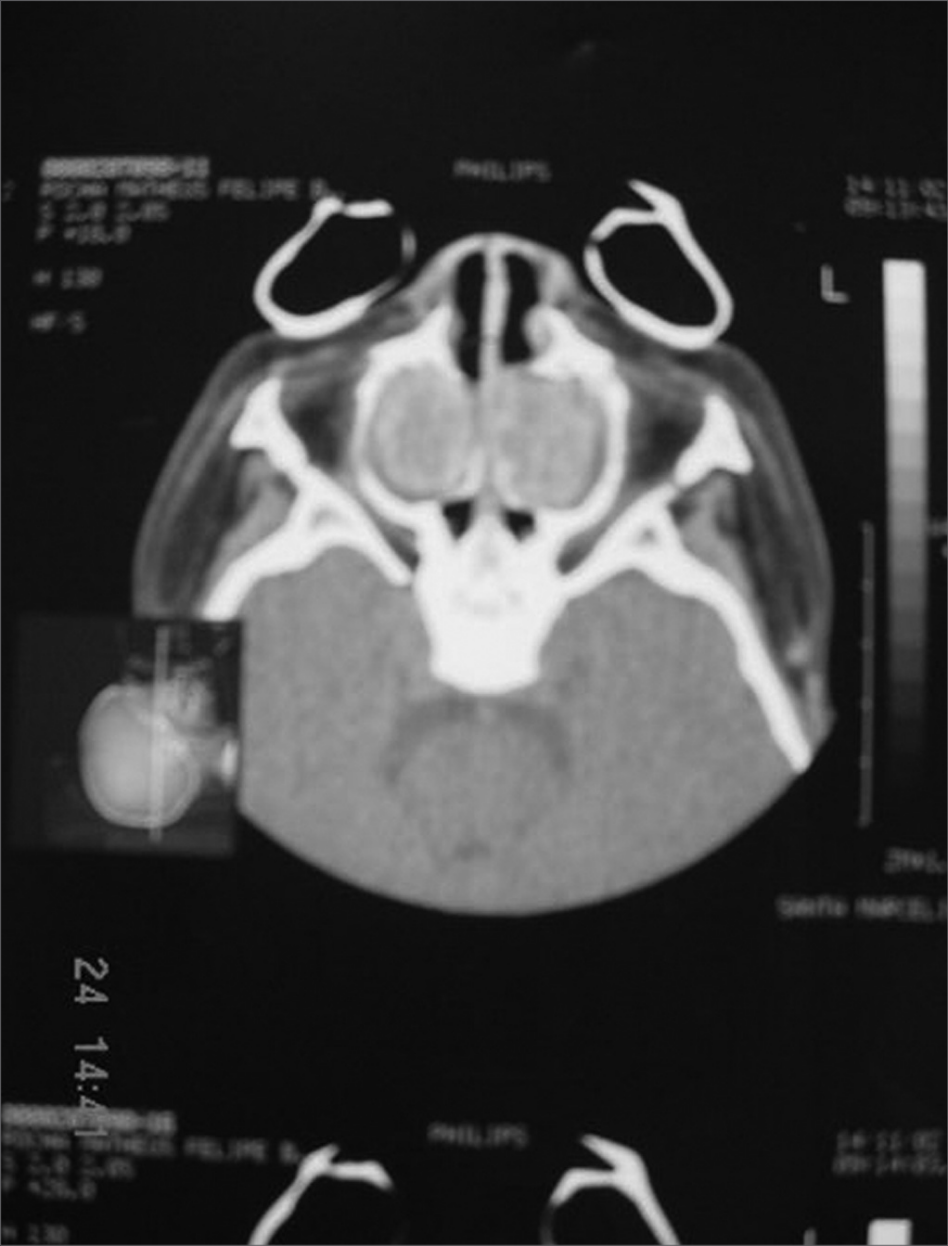
Paranasal sinuses CT Scan, axial view, soft tissue window, showing a cystic-type lesion with a peripheral halo around the maxillary sinuses, emphasizing a chronic inflammatory process. Such cystic formation causes the bulging of the lateral nasal wall and nasal cavity narrowing.

**Figure 3:**
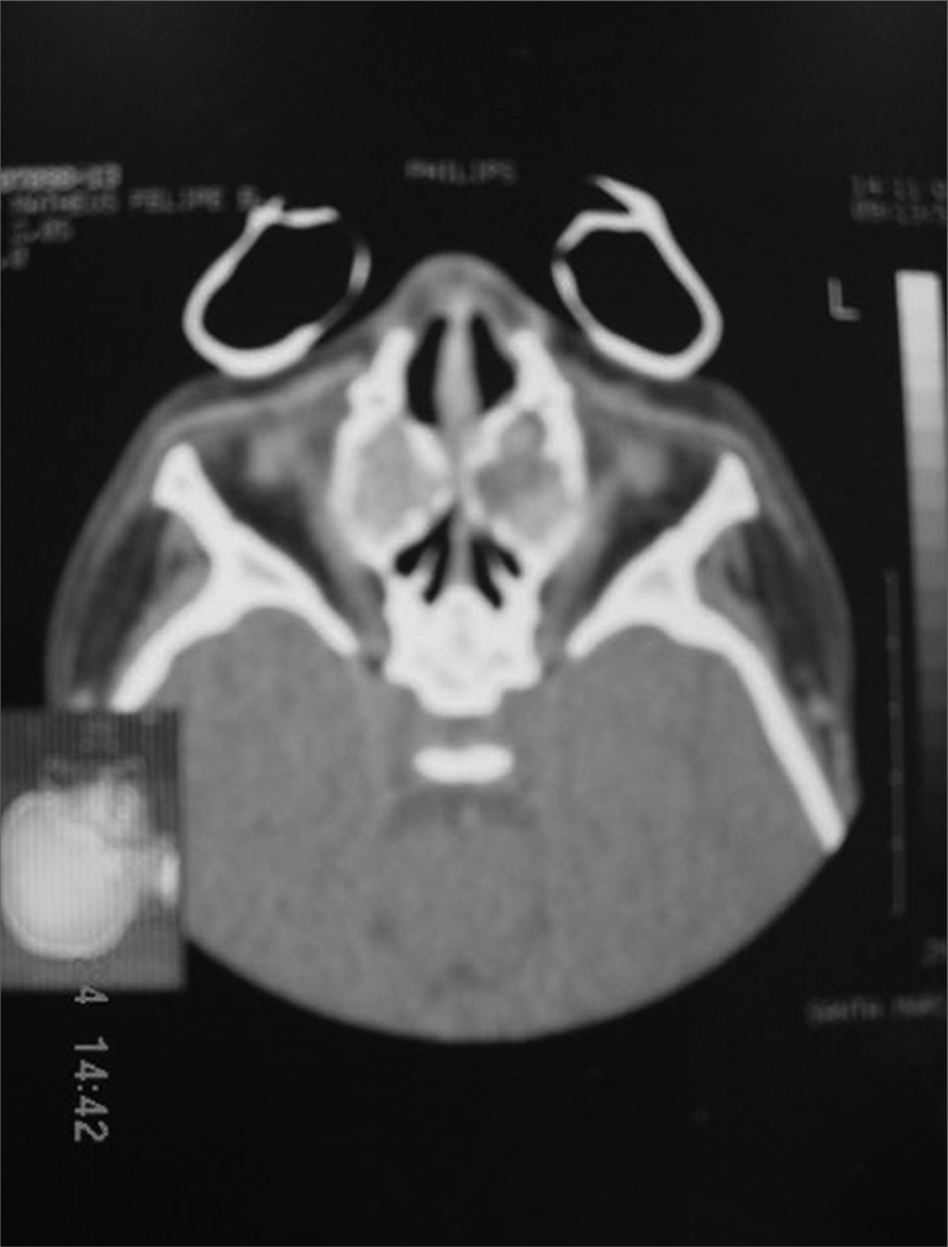
Paranasal sinuses CT Scan, axial view, bone window, showing nasal cavities obstruction caused by a bilateral maxillary cystic formation and a hypertrophy of pharyngeal tonsils.

The literature is full of controversies as to the terminology of this new sinusal pathological entity, more broadly studied when CT Scans became part of the investigative weaponry of paranasal sinuses. Some authors use the term “maxillary mucocele”^10^. Other authors have found purulent collections and caseous material during surgery, thus preferring the term “mucouspiosinusitis”.”^4^ However, the most adequate term for our patient in question is “pseudomucocele”, characterized by the finding of a viscous secretion inside the paranasal sinuses, causing images of central hyperdensity and peripheral hypodensity images on CT Scan. It is not a cystic lesion with true epithelial walls, but rather, the mucous secretion is limited by a capsule of inflammatory tissue that follows the shape of the sinuses walls.^8^

Literature shows some interesting aspects regarding pseudomucoceles. Its etiopathology still remains obscure. Some papers try to correlate its formation with glandular alterations, transport alterations within the cell and at a molecular level, genetic expressions and the very presence and action of inflammatory mediators.^10^ The largest incidence of this disease occurs among younger patients^4^, as seen in our patient here. Older patients have a greater incidence of nasal polyps^9^. The maxillary sinus is the one most frequently affected. Ethmoid involvement is more variable. Maxillary sinus anatomy impairs its drainage, causing obstruction and the retention of secretions with a reactional mucosal edema.^10^

In our patient, subject of the present study, we noticed the findings frequently mentioned in the literature, such as lateral nasal cavity walls bulging and the presence of mucous secretion. In adults and older children, bone alterations are characterized by erosions caused by chronic inflammatory processes or mucopiosinusitis.^49^

Sodium and chlorine concentrations in sweat were measured in order to confirm the suspicion of cystic fibrosis; however, image and clinical findings strongly suggest the diagnosis. The sweat test is considered the gold Standard for the diagnosis of cystic fibrosis, and chlorine values above 60mEq/L are accepted for the diagnosis of cystic fibrosis. Patients presenting values between 40-60mEq/L must be followed up, and confirmation is done by means of a genetic study. Sweat test must be repeated in different days, since there is the possibility of attaining false-positive or false-negative results, especially when the genetic study does not confirm the diagnosis ^12^.

As far as treatment is concerned, there is a trend to have a more conservative approach, with antibiotic agents, steroids and frequent nasal washings, since some studies that follow these patients up have reported on a trend for pseudomucocele spontaneous clinical resolution10. Thus, not all the children affected by this disease need to undergo surgery. The choice for a surgical approach together with clinical treatment in this hereby reported case was based on the severity of his clinical condition, such as severe nasal obstruction, sleep alterations, height and weight development retardation, and the major repercussion in his lower respiratory tract, which was reported in the history of recurrent pulmonary infections and repeated hospital stays, with the risk of him acquiring hospital infections. Even with proper treatment, symptom recurrence is common, as is nasosinusal infection by Pseudomonas sp, which may facilitate lower respiratory tract infections. Notwithstanding, some authors prefer clinical treatment followed by nasal endoscopic surgery in all cases that complicate with nasosinusal conditions in cystic fibrosis patients who undergo lung transplant. Davidson et al. indicate a rigorous regimen of nasal washings with saline solution in order to fluidize secretions, as well as nasal flushing with Tobramycin daily in order to inhibit the growth of pseudomonas.^11^ Our approach in the post-operative period has been to keep on doing frequent nasal flushings with isotonic saline solution. The patient improved significantly from the nasal obstruction, without signs and symptoms of pseudomucocele recurrence during the three month observation period.

## CONCLUSIONS

Pseudomucoceles have become part of the differential diagnosis routine since CT Scans came along to help in the investigation of sinusal diseases. It is a rare entity which is closely related to nasosinusal manifestations of cystic fibrosis.

Clinical signs strongly suggest the diagnosis of cystic fibrosis, leading the specialist to investigate this disease through CT scans, laboratory and genetic studies.

Patients with pseudomucoceles have enjoyed a relevant increase in survival, thanks to current treatment modalities.

Recurrences of the common manifestations of such disease and its complications may suggest more rigorous indications for surgical approaches and the long term follow up of these patients.
